# Adaptive On-the-Fly Changes in Distributed Processing Pipelines

**DOI:** 10.3389/fdata.2021.666174

**Published:** 2021-11-26

**Authors:** Toon Albers, Elena Lazovik, Mostafa Hadadian Nejad Yousefi, Alexander Lazovik

**Affiliations:** ^1^ Monitoring & Control Services Department, TNO, Groningen, Netherlands; ^2^ Distributed System Group, Faculty of Science and Engineering, Bernoulli Institute, University of Groningen, Groningen, Netherlands

**Keywords:** distributed computing, big data applications, on-the-fly updates, adaptive dynamic systems, industrial data management, dynamic software updating

## Abstract

Distributed data processing systems have become the standard means for big data analytics. These systems are based on processing pipelines where operations on data are performed in a chain of consecutive steps. Normally, the operations performed by these pipelines are set at design time, and any changes to their functionality require the applications to be restarted. This is not always acceptable, for example, when we cannot afford downtime or when a long-running calculation would lose significant progress. The introduction of variation points to distributed processing pipelines allows for on-the-fly updating of individual analysis steps. In this paper, we extend such basic variation point functionality to provide fully automated reconfiguration of the processing steps within a running pipeline through an automated planner. We have enabled pipeline modeling through constraints. Based on these constraints, we not only ensure that configurations are compatible with type but also verify that expected pipeline functionality is achieved. Furthermore, automating the reconfiguration process simplifies its use, in turn allowing users with less development experience to make changes. The system can automatically generate and validate pipeline configurations that achieve a specified goal, selecting from operation definitions available at planning time. It then automatically integrates these configurations into the running pipeline. We verify the system through the testing of a proof-of-concept implementation. The proof of concept also shows promising results when reconfiguration is performed frequently.

## 1 Introduction

Industrial organizations are increasingly dependent on the digital components of their business. Industry 4.0 is based on further digitalization and, in particular, on the concepts of automation and data exchange to achieve efficiency and zero-downtime manufacturing. Organizations trying to keep pace with the new challenges are faced with processing a large number of data (Che et al., 2013). It is required for their core business and it becomes a part of their decision-making processes. Additionally, it provides a competitive advantage over companies not investing in digitalization. To achieve their goals, industrial organizations must often deal with various kinds of data. This leads to different requirements regarding how that data is processed. For example, some data such as readings from physical sensor networks from factory equipment may require real-time processing, while other data such as customer or supplier analytics can be processed in batches at set intervals (Assunção et al., 2015).

Performing analysis on all available datasets on a single computer may not be fast enough or may be impossible due to the operational infrastructure requirements (such as storage space or memory). By distributing the processing over multiple computers, the hardware requirements per computer can be decreased and total processing time can also be lowered as each computer operates in parallel. While taking a mainframe approach (i.e., a single high-performance computer) may be possible, it is often not as cost-effective as a distributed approach (Franks, 2012). Such distributed processing and analysis are commonly done through *distributed processing pipelines*. Note that the term *pipeline* is sometimes also used to describe machine learning systems built on top of distributed data processing frameworks. Machine learning specific aspects are not covered in this paper. We define a pipeline as generic distributed data processing performed through a sequence of steps, where each step performs a specific part of data processing, and the output of that step is used as an input to one or multiple subsequent steps.

The distributed data processing in Industry 4.0 has a number of open issues. One of these is the fixed nature of distributed processing platforms currently available on the market. That means that steps in a pipeline on one of these platforms cannot be changed once a calculation has been started. However, updating a running pipeline is needed in many cases including the following:

• After changes in the environment
  e.g., external services become unavailable or a new data source provides data in a different format.
• After changes in business model and goals
  e.g., calculating different statistics based on the same data as a result of business industrial demands.
• Upgrading of processing models or parameters
  e.g., fixing errors in the pipeline code to provide new functionality, introducing more accurate algorithms, or tweaking and tuning algorithm parameters for better results.

Two approaches are generally used to update distributed processing pipelines^1^. The first requires stopping a running pipeline and then starting a new updated version. It is not always appropriate or possible, such as in the case of permanent monitoring and controlling systems that need to be operational 24/7 or for batch processing pipelines that are in the middle of a long-term computation. In these cases, we cannot always afford the resulting downtime or loss of progress, or it could simply not be desirable. The second option is executing a new updated version in parallel with the old version and taking over processing when the new pipeline is ready. If the processing resources required for a pipeline are significant, running a new pipeline in parallel is not always an option because of the limited infrastructure available or excessive extra costs required for it.

In the case of stopping and then restarting, the resulting downtime could delay or completely miss the analysis of vital data. In the case of parallel computations, significant progress could be lost, depending on how long ago the computation had been started.

In a previous paper, we have developed a framework *spark-dynamic* (Lazovik et al., 2017), built on top of the popular distributed data processing platform Apache Spark (The Apache Software Foundation, 2015b) to enable the updating of the steps and algorithm parameters of running pipelines without restarting them. This process is called *reconfiguration*. In this work, we extend the functionality of *spark-dynamic* to automate parts of the reconfiguration process using Artificial Intelligence Planning techniques to guarantee consistency of performed updates. The resulting system uses constraints of different types to model pipeline behavior. It is able to automatically generate and validate pipeline configurations based on the provided model and goals and can automatically integrate these configurations into a running pipeline, even when internal pipeline data types differ between versions.

The need for verified reconfiguration is twofold. First, the *spark-dynamic* library only provides a basic updating mechanism with checks for serialization success and type compatibility. However, this is not enough, because, for example, changes to the internal functionality of one step could differ from the expectations of some subsequent step, which could result in general inconsistencies or outright crashes of the whole process. Secondly, the verification process is a complex task, and by automating some aspects of the reconfiguration process, we can drastically simplify it. In the future, this may allow industrial users without any development experience to make changes to a running pipeline when they are required without coding efforts. For example, an asset manager who wants insight into the state of equipment and the trends in aging of that equipment can start such a permanent analysis, check the results at any moment, and tweak the business or technical constraints when it is needed. What is important, though, is that when the update happens, the end user should have enough trust that it does not crash the whole system. Automated synthesis and validation of every update allow us to formally ensure that trust.

Many distributed data processing frameworks operate on the principles of a Directed Acyclic Graph (DAG), distinguishing themselves through their focus on batched or streaming data processing [e.g., Apache Spark (The Apache Software Foundation, 2015b) mainly focuses on batched processing while supporting streaming workloads, whereas for Apache Flink (The Apache Software Foundation, 2015a), the opposite is true]. In this paper, we use Apache Spark as the distributed data processing framework due to its popularity. However, as many modern frameworks are built on similar principles, the mechanisms described in this paper are in major parts applicable to other distributed data processing frameworks.

**FIGURE 1:**
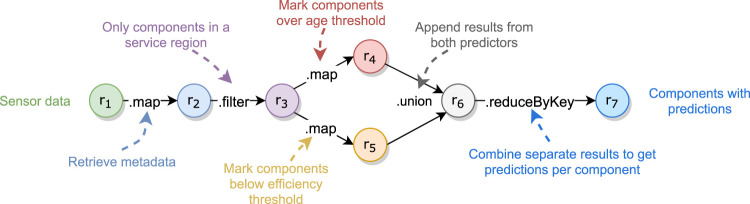
Example predictive maintenance pipeline.

A typical example of a distributed data processing pipeline for Industry 4.0 is the case of predictive maintenance with the goal of zero downtime. With predictive maintenance, factories and other industries can improve the efficiency of their systems and prolong their lifetime. Consider a goal of finding devices in a factory that degrade over time. With predictive maintenance, we could find degraded devices before they fail. Predictors could be the age of devices or the measured efficiency through some sensors. A schematic Spark data pipeline can be constructed as shown in Figure 1.

Later, new predictors could be developed such as one based on the failure rate of each type of device. Existing predictors could also be found ineffective and be removed from service.

In summary, the main contribution of this paper is a distributed data processing pipeline reconfiguration framework based on constraint-based AI planning. It ensures that the current industrial user goals are satisfied, takes into account the dependencies between related steps within the pipeline (and thus ensuring its data type and structural consistency), and automatically incorporates the new configuration. The feasibility of the approach is tested using Apache Spark as a target distributed processing framework. In this paper, we further demonstrate the generic methodology to enable adaptive on-the-fly changes of applications in distributed data analysis for industrial organizations in the Industry 4.0 era and a software library as a proof of concept with the demonstration of the guarantees of updating for the industrial user.

The rest of the paper is organized as follows. In Section 2, we look at current research into runtime updating, pipeline synthesis, and consistency checking. Then, in Section 3, we show an overview of our proposed system. This is followed by a closer look at the planner design in Section 4. Next, we provide an evaluation of the system in Section 5. Finally, we provide conclusions and discussion in Section 6.

## 2 Related Work

The problem of distributed software reconfiguration is not new. We, therefore, start with an insight into the state of the art of relevant techniques. We begin by looking at dynamic updates not specific to distributed processing.

### 2.1 Runtime Updating

A common term for runtime updating is Dynamic Software Updating (DSU) (Hicks and Nettles, 2005; Pina et al., 2014). With DSU, running processes are updated by rewriting the running code and process memory. Compared to traditional software updating, the process does not need to be stopped, although possibly it can be temporarily halted. Because of this, the state of the running process can be preserved. As a result, running sessions and connections can be kept active and no costly application boot is required.

Many early updating systems require a specialized program environment. Notably, Cook and Lee (1983) have described a system called DYMOS that encompasses nearly all aspects of a software system: a command interpreter, a source code manager, an editor, a compiler, and a runtime support system. By having control over all of these aspects, they have the ability to add and monitor synchronization systems allowing the updates to be performed seamlessly.

More recent models for DSU do not require an all-encompassing system. Instead, DURTS (Montgomery, 2004) requires only a custom linker and a module to load and synchronize replacement modules. The linked module is loaded into heap space from within the application and a pointer-to-function variable is used to execute the function, where the pointer value is updated to point to newer versions. Similarly, Hicks and Nettles (2005) have described an approach where they used the C-like language *Popcorn*, compiling code patches into *Typed Assembly Language* that can be dynamically linked and integrated. There are other works on a language level like (Mugarza et al., 2020), which are implemented on the *Ada* programming language. Alternatively, Bagherzadeh et al. (2020) present a language-independent approach based on model execution systems (Hojaji et al., 2019).

Some other systems use and extend the functionality provided by the platform an application runs on. For example, Kim et al. (2011) used the Java Virtual Machine (JVM) HotSwap capability to replace code, adding features such as bytecode rewriting to work around HotSwap limitations. One of the more complex systems is Rubah (Pina et al., 2014). It uses a manual definition of update points, combined with bytecode rewriting. Their update process consists of three steps. The first step is *quiescence*, reaching a stable state where it is safe to perform updates. In the next step, the running state is transformed, going over the objects in heap memory and changing the fields and methods from existing objects to their new versions. Next, the program threads are restarted at their equivalent location in the new version of the application. Later, Pina et al. (2019) improved system availability by warming up updates. They run old and new versions and perform the update if both versions converge. Neumann et al. (2017) and Gu et al. (2018) later introduced similar systems based on the same principles. Šelajev and Gregersen (2017) presented a runtime state analysis system to detect runtime issues caused by updating Java applications.

Clearly, many different DSU systems and types of systems exist. In fact, as early as 1993, Segal and Frieder (1993) have given a summary of on-the-fly updating. Some solutions require a complete restructuring of code to support updates and some even act as an entire operating system. Seifzadeh et al. (2013) have provided a more recent overview of different dynamic software update frameworks and approaches. They have also included a categorization for these updating frameworks and describe the metrics by which the frameworks are then compared. Mugarza et al. (2018) also analyzed the existing DSU techniques focusing on safety and security.

Most approaches require complete control over the environment in which the software is executed. However, developing applications in distributed systems is much more complicated, because process control has been handed to distributed data processing platforms such as Apache Spark (Zaharia et al., 2010), Flink (Carbone et al., 2015), and Storm (Toshniwal et al., 2014) instead of the user code. The platforms handle distribution, scheduling, and execution automatically, and users have only marginal influence in these areas. Implementing the approaches described above would require changes to the distributed processing platforms. However, modifying the existing distributed stream processing frameworks is undesirable as they are meant to act as a general core, where the user applications simply use existing functionality. Solutions should then be found that work within the current control paradigms to also work with the newer version of the application.

### 2.2 Updating Distributed Data Processing Pipelines

Updating variables of a pipeline is a common way of adding flexibility to distributed pipelines. Boyce and Leger (2020) provided a solution on top of Apache Spark for changing variables at the runtime by extending Broadcast variables. In Apache Flink, one can use the CoFlatMapFunction to get two streams of the original data and the parameters streams and assign parameters for each data record or use Apache Zookeeper (Hunt et al., 2010) for storing the configuration and let the Apache Storm application listen for an update. However, all these approaches have two significant limitations, 1) only the parameters can be updated; 2) the updates should be anticipated before launching an application.

Another approach is to use variation points as originally defined for Software Product Lines (SPLs) (Pohl et al., 2005). SPL is a concept where reusable components are created for a domain that can then be composed in multiple ways to develop new products. However, the variation points still need to be defined before launching the software. Overcoming this issue, Dynamic Software Product Lines (DSPLs) (Hallsteinsen et al., 2008; Eichelberger, 2016) extend SPL to allow the composition of predefined components to be done at runtime.

To apply DSPLs to distributed data processing pipelines, we must first be able to model such pipelines. Berger et al. (2014) and Dhungana et al. (2014) have described approaches to model topological variability, by which they mean connecting components in a specific order and in interconnected hierarchies. These hierarchies then have to be respected during reconfiguration. Qin and Eichelberger (2016) have previously implemented DSPLs on top of Apache Storm, allowing runtime switching between alternatives, but requiring that they already be implemented at design time.

### 2.3 Spark-Dynamic

In a previous work done by the authors (Lazovik et al., 2017), we have investigated the feasibility of dynamically updating the processing pipeline of an Apache Spark application. Apache Spark is one of the most popular big data processing platforms. It is a unified engine providing various operations, including SQL, Machine Learning, Streaming, and Graph Processing. Spark is based on the concept of Resilient Distributed Datasets (RDDs). An RDD is a read-only, distributed collection partitioned into distinct sets distributed over a computing cluster. RDDs form a pipeline which is a DAG where performing an operation over one RDD results in a new RDD. Edges of the Spark DAG are standard operations, e.g., map and reduce, while inside each operation, there is a user-defined function. Internally, the Spark task scheduler uses *scopes* instead of RDDs, where a single operation is represented by one scope but may internally create multiple (temporary) RDDs.

The *spark-dynamic* framework is an extension on top of Apache Spark, making it able to update both parameters and functions within pipeline steps during runtime. Variation points can be updated using a REST API, where functions are updated by providing a new byte code. Instead of the fixed alternatives of Qin and Eichelberger (2016), new algorithms and new version of algorithms can be used. The extension wraps each operation to pull the updated value for parameters and functions on every invocation. The wrapped methods are named dynamic(Operation) where operation is the original method name. For example, dynamicMap is a wrapper for the map operation.

Apart from updating parameters and functions, *spark-dynamic* can also change data sources on the fly. An intermediate Data Access Layer is introduced to intervene between the Spark processing pipeline and Spark Data Source Relation, which is responsible for preparing the RDD for the requested data.

The performance of the prototype was also measured as part of the feasibility study, with promising results (Lazovik et al., 2017). The solutions from this paper are applied on top of this earlier system.

### 2.4 Techniques for Building and Checking Pipelines

With research showing the feasibility of modeling and updating distributed processing pipelines, given a distributed computational pipeline with placeholders, we should also be able to automatically select a component for each placeholder to satisfy the goal of the pipeline. To ensure that the newly generated pipeline configuration is valid and, for a running pipeline, that parameter updates do not introduce any errors, we must apply some form of consistency checking. When developers want to introduce an update, they are able to change both objects and functions as long as their signatures stay the same. However, these signatures do not describe all details of these updates. For example, a function may take the same types and number of arguments and yet provide different results. Consider *f*(*a*: *Double*, *b*: *Double*) → *a*∗*b* and *g*(*a*: *Double*, *b*: *Double*) → *a*/*b* that share the same signature but should be used differently. Since only signature checking is not enough, we must research other methods of consistency checking. We have focused on the topics of model checking, constraint programming, and automated planning since these techniques are relatively popular (Ghallab et al., 2004) and extensible and do not require the use of complex features such as a cost function or probability calculations.

Model checking is a brute-force method of examining all possible states of a system (Baier and Katoen, 2008), to determine if, given a program *M* and a specification *h*, the behavior of *M* meets the specification *h* (Emerson, 2008). The program is represented in specialized languages such as PROMELA or through analysis of source code such as C (Merz, 2001). The specification can among others be done through Linear Temporal Logic (LTL) and Computation Tree Logic (CTL).

We could use model checking to verify if a proposed pipeline configuration is valid. For the generation of configurations, however, we would need to brute-force the search space; i.e., we would need to iterate over every possible configuration until one is found that satisfies the property specification. This method of evaluation is not very efficient and therefore we would also have to investigate heuristics to speed up the process.

In constraint programming, the behavior of a system is specified through constraints, for example, by restricting the domains of individual variables or imposing constraints on groups of variables (Bockmayr and Hooker, 2005). This is done by having each subsequent constraint restrict the possible values in a constraint store. This way all possible combinations can be tested and a solution can be given if all constraints can be met. Basic constraints exist such as *v*_1_.gt(*v*_2_) that defines an arithmetic constraint over two variables *v*_1_ and *v*_2_, as well as global constraints such as model .allDifferent(*v*_1_,…,*v*_*n*_) (van Hoeve, 2001) that are defined over sets of variables.

Automated planning is a relatively broad subject, but classical planning is perhaps the most general. Classical planning is based on transition systems. States are connected through transitions, in which an action is applied that actually changes the one state into a consecutive one (Ghallab et al., 2004). An action typically has preconditions and effects. The preconditions are propositions required on a state to be able to apply the action, and the effects are the propositions set on the resulting state. Note that, in literature, an action is typically defined as a ground instance of a planning operator or action template (Ghallab et al., 2016). These operators or templates can include parameters that define the targets on which the preconditions and effects must apply. For simplicity, we will only reason about grounded planning operators in this work, and as such we will use only the term action instead of planning operator. Planning aims to solve a planning problem, which often consists of the transition system, one or more initial states, and one or more goal states.

Different types of transition systems are used, one of which is State Transition Systems (STSs) (Ghallab et al., 2004; Ghallab et al., 2016). In this model, states can be changed not only by actions but also by events, which cannot be controlled. They are defined as Σ = (*S*, *A*, *E*, *γ*), with *S* being the set of states, *A* being the set of actions, *E* being the set of events, and the state transition function *γ*: *S* × (*A* ∪ *E*) → 2^*S*^ (Nau, 2007). Further, restricted STSs do not allow events; thus, Σ = (*S*, *A*, *γ*). In this case, Ghallab et al. (2016) define the transition function as *γ*: *S* × *A* → *S* since there is only one resulting state for a transition. A planning problem on such a system is defined as $P=\left(\Sigma ,s_{0},g\right)$ , with *s*_0_ being the initial state and *g* being the set of goal states.

Ghallab et al. (2004) distinguish three representations of classical automated planning: set-theoretic representation, classical representation, and state-variable representation.

In the set-theoretic representation, each state is a set of propositions, and each action has propositions that are required to apply the action (preconditions), propositions that will be added to a new state when the action is applied (positive effects), and propositions that will be removed (negative effects). The classical representation is similar to the set-theoretic representation, except states are logical atoms that are either true or false, and actions change the truth values of these atoms. In the state-variable representation, each state contains the values for a set of variables, where different states contain different values for these variables and actions are partial functions that map between these tuples.

These base techniques are often extended. For example, some authors describe the use of model checking to solve planning problems (Cimatti et al., 1998; Giunchiglia and Traverso, 2000). Similarly, Kaldeli (2013) describes a planner built through constraint programming, based on earlier work from Lazovik et al. (2005), Lazovik et al. (2006). The planner definitions such as the actions and goals are first translated into constraints. Then, the constraints are evaluated using an off-the-shelf Constraint Satisfaction Problem (CSP) solver.

When comparing model checking and automated planning, both techniques have the same time complexity (EXPTIME or NEXPTIME for nondeterministic planning) (Ghallab et al., 2004; Meier et al., 2008). However, instead of the brute-force validation of configurations when using model checking, automated planning can use heuristics to reduce the search space, which could decrease the final planning time. Furthermore, automated planning techniques use proven concepts for the generation of plans which could serve as a basis on top of which we define our pipeline concepts, whereas for model checking, the basic configuration generation algorithms would still need to be designed.

Finally, we compare constraint programming and automated planning. The automated planning concepts are more abstract, separating the planning goal from the state and transition behaviors. Planning has a solid foundation with its transition systems, while constraint programming permits a lot of freedom in the behavior that can be built using constraints. Instead of choosing between these two techniques, we can combine them. We can use constraint programming as a basis to create an automated planner, by implementing the transition systems and other planning concepts using constraints. We then retain the flexibility of constraint programming in case we want to add features to the planner later.

## 3 General Overview

**FIGURE 2:**
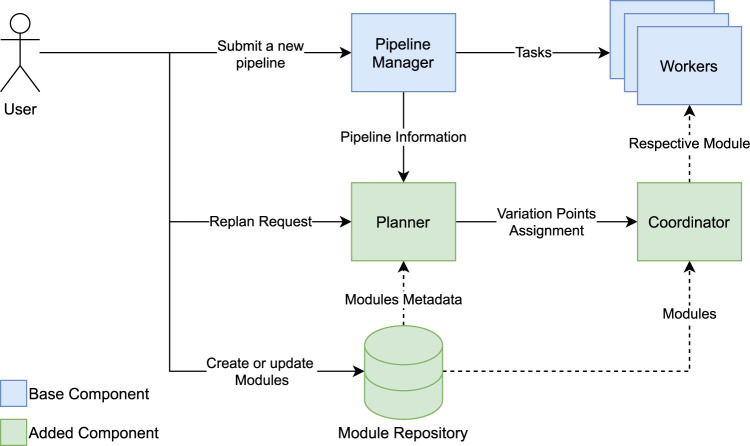
Conceptual overview of the system.

We designed a system to help the designer plan a pipeline while supporting runtime updates in distributed systems. The proposed system is depicted in Figure 2. There are three types of user activities: I) submit a new pipeline, II) create or update modules, and III) request replanning the pipeline during runtime. Our design is platform-independent and could be implemented on top of various distributed data processing platforms. The blue components are the bases of almost every such platform, and the green ones are the extra components that we proposed and could be seen as plugins to the existing platforms.

The user does almost everything the same as before, which was developing the code for the designed pipeline describing what each step does and how they are connected. However, we help the user in deciding what each step should do to reach the goal. The user can create a pipeline where the steps of the pipeline contain calls to variation points instead of directly containing user code. The code referenced from the variation points is not fixed and can be updated at runtime. The user can also annotate the pipeline with constraints, such as specifying initial conditions and goals. Finally, the user submits the pipeline code to the pipeline manager.

Apart from the pipeline, the user also submits code that can be executed from the variation points. This code is prepared as a PlanningModule, which contains the user code as well as metadata such as the function signature and user constraints. These constraints describe the functionality of the module in such a way that the planner can determine if the module is needed to fulfill a pipeline’s goal.

After submission, the pipeline manager compiles the pipeline code into tasks and pipeline information. The tasks are directly related to pipeline steps which may have fixed functionalities or contain a variation point. The pipeline manager then distributes the tasks among the cluster of workers to execute the tasks. The pipeline manager and workers can be mapped to any platform. For example, they can be a Spark Driver and Workers or Hadoop JobTracker and NodeManagers. Simultaneously, the pipeline manager submits a planning request to the planner. The request contains the pipeline information describing the steps, inputs, desired output, and variation points.

The planner then decides on the modules that need to be placed at each variation point in the pipeline to meet the goals. If there is a feasible assignment, the planner will send it to the coordinator.

At the same time, the workers start executing tasks. For each execution of variation points, the workers will request the coordinator to hand them the respective module to run. The coordinator will then fetch the module from the repository according to the planner assignment or wait until a plan is available. The workers and the coordinator continue to run the pipeline collaboratively, where the worker executes the modules provided by the coordinator for each variation point.

The module repository contains PlanningModules and their different versions. Note that the assignment contains the modules’ versions, and the coordinator will continue to use the assigned version unless a new assignment arrives.

After the pipeline has started, the user can also manually request the planner to update the assignments. The planner will do the same as before while also fetching the new and updated modules from the repository. If the planner finds a new feasible assignment, it will inform the coordinator about the updates; otherwise, the assignments will remain intact. Any updates to the assignments of variation points must go through the planner to ensure that the pipeline is consistent and the update will not break it.

## 4 Planner Design for Pipeline Reconfiguration

The planning process described in this section performs two roles at once, both generating and validating configurations. If we represent the planning problem using constraints, any valid assignment of variables that satisfies all constraints can be regarded as a valid plan. Given an encoding of the planning problem as a CSP, the constraint solver would be able to perform the planning process by attempting each possible configuration of actions. We use classical planning techniques as they have been shown to be appropriate for restricted STSs. We use the state-variable representation for its expressiveness as well as the similarity of concepts with constraint programming.

### 4.1 Core Planning Model

Before we describe how to transform our pipeline information into a planning problem, we must first formulate the problem of pipeline reconfiguration as a planning problem.

We modify the classical definitions presented by Ghallab et al. (2004); since our planning problem does not contain a single initial state, our transition system can contain branches and joins and our plans must match the topology of the Spark pipeline we are planning for. Thus, we define our planning problem as

$$P=\left(\Sigma ,\overset{≃}{\Sigma },S_{0},S_{g}\right)$$ (1)

With the transition system Σ = (*S*, *A*, *γ*) and with $\overset{≃}{\Sigma }$ to map the planning problem to our domain, we simplify the notation *s*_*m*_ ∈ *γ*(*s*_*n*_, *a*), that is, the application of action *a* onto *s*_*n*_ resulting in *s*_*m*_, as $s_{n}\overset{a}{\to }s_{m}$ . Each state in *S* is represented by state variables; i.e.,

(∀s∈S):s={v∈Vars|(v,val(s,v)⊆Dom(v))} (2)

Here, *Vars* is the set of variables in our planning problem, *Dom*(*v*) is the domain of a specific variable *v* (i.e., all possible assignments to an instance of that variable), and *val*(*s*, *v*) represents the value or possible values of the state-variable representing variable *v* in state *s*. *S*_0_ ⊆ *S* is the set of initial states and *S*_*g*_ ⊆ *S* is the set of goal states. Note also that there are no states before the initial states, i.e., (*∀s*_0_ ∈ *S*_0_)(*∀s*_*n*_ ∈ *S*)(*∀a* ∈ *A*): *s*_0_∉*γ*(*s*_*n*_, *a*), and there are no states after the goal states, i.e., (*∀s*_*g*_ ∈ *S*_*g*_)(*∀a* ∈ *A*): *γ*(*s*_*g*_, *a*) = ∅.

As in Ghallab et al. (2004), we define an action as

a=(name(a),precond(a),effects(a)) (3)

Supported preconditions and effects are variable equality (both with constant or other variables), constraint conjunction, constraint disjunction, and the negation of these constraints [e.g., “(a := 1) &:& (b !:= true)”]. Similar to (Kaldeli, 2013), each constraint is a propositional formula over a state variable or a combination of two constraints. To apply $s_{n}\overset{a}{\to }s_{m}$ , we must ensure that *s*_*n*_ satisfies all preconditions of the action and that the effects of the action can be applied onto *s*_*m*_. We can encode this with a generalized “*state* satisfies *constraints*” relation, where *s*_*n*_ satisfies *precond*(*a*) and *s*_*m*_ satisfies *effects*(*a*). We define this relation, using *Cstrs* as the set of all constraints in the planning problem, as

(∀c∈Cstrs):satf(state,c) (4)

$$\begin{array}{l} satf\left(s,constraint\left(a,op,b\right)\right)=\\ \;\left\{\begin{array}{cc} \;\left\{\begin{array}{cc} \;val\left(s,a\right)=b,\; & \text{if }a\in Vars\wedge b\in Dom\left(a\right)\\ \;val\left(s,a\right)=val\left(s,b\right),\; & \text{if }a\in Vars\wedge b\in Vars \end{array}\right.\; & \;eq\\ \;\left\{\begin{array}{cc} \;val\left(s,a\right)\neq b,\; & \text{if }a\in Vars\wedge b\in Dom\left(a\right)\\ \;val\left(s,a\right)\neq val\left(s,b\right),\; & \text{if }a\in Vars\wedge b\in Vars \end{array}\right.\; & \;neq\\ \;\left\{\begin{array}{cc} \;satf\left(s,a\right)\wedge satf\left(s,b\right),\; & \;\text{if }a\in Cstrs\wedge b\in Cstrs \end{array}\right.\; & \;and\\ \;\left\{\begin{array}{cc} \;satf\left(s,a\right)\vee satf\left(s,b\right),\; & \;\text{if }a\in Cstrs\wedge b\in Cstrs \end{array}\right.\; & \;or\\ \;\left\{\begin{array}{cc} \;\neg satf\left(s,a\right)\vee \neg satf\left(s,b\right),\; & \;\text{if }a\in Cstrs\wedge b\in Cstrs \end{array}\right.\; & \;nand\\ \;\left\{\begin{array}{cc} \;\neg satf\left(s,a\right)\wedge \neg satf\left(s,b\right),\; & \;\text{if }a\in Cstrs\wedge b\in Cstrs \end{array}\right.\; & \;nor \end{array}\right. \end{array}$$ (5)

where a constraint is described by a source *a*, a target *b*, and an operation *op*, with *op* translated from a constraint as defined by Table 1.

**TABLE 1:** Mapping between constraints and their representation in the planning problem as *op*.

Op	Constraint	Constraint type
eq	a =:= b	Precondition
	a := b	Effect
neq	a !:= b	Precondition
	a !:= b	Effect
and	a &:& b	Both
or	a|:|b	Both
nand	(not) a &:& b	Both
nor	(not)a|:|b	Both

We also formulate the *frame axiom* similar to Barták et al. (2010) and Kaldeli (2013), which specifies that, unless the action being applied on a state modifies a state variable, the state variable will remain the same in the state following that action:

$$\begin{array}{cc} \left(\forall s_{n}\in S\right)\left(\forall s_{m}\in S\right) & \left(\forall v\in Vars\right)\left(\forall a\in A\right):\\ s_{m}\in \gamma \left(s_{n},a\right)\;\Rightarrow & val\left(s_{n}\right),v=val\left(s_{m},v\right)\\ & \vee vaffectedByAction\left(a\right) \end{array}$$ (6)

vaffectedByAction(a)≡(∃c∈effects(a)):caff(v) (7)

$$\begin{array}{l} constraint\left(a,op,b\right)aff\left(v\right)=\\ \left\{\begin{array}{cc} a=v,\; & \text{if }a\in Vars\\ b=v,\; & \text{if }b\in Vars\\ aaff\left(v\right)\;\vee \;baff\left(v\right),\; & \text{if }op\in \left\{and,or,nand,nor\right\}\\ false,\; & \text{otherwise} \end{array}\right. \end{array}$$ (8)

With the base planning model fully described, we extend it below to support the planning of pipelines.

### 4.2 Mapping to the Distributed Pipeline

Since the plans we generate must be applied onto a fixed Spark pipeline topology, we cannot simply generate any plan that satisfies the goal constraints. Instead, we must relate our STS (*State* → *Action* → *State*) to the Spark DAG (*Scope* → *Operation* → *Scope*). To implement this mapping, we first construct a separate transition system to which the final plan must be isomorphic. That is, any relation in the STS should be represented in the DAG and vice versa. We define this isomorphic system as

$$\overset{≃}{\Sigma }=\left(R,T,rdd,VP,vp,\overset{≃}{\gamma }\right)$$ (9)

We describe the parts of this equation in this section. The set *R* represents the RDD *scopes* in the pipeline, and *T* represents the Spark operations that transform one RDD scope to a new scope. The transition function $\overset{≃}{\gamma }:R\times T\to R$ describes how the transitions apply to the RDD scopes.

Besides adding constraints to actions, we also allow constraints to be added to the pipeline topology itself, both manually by the user and automatically inferred from the pipeline topology, such as the datatype of the initial RDD. Preconditions added by the user are applied to the scope they are defined on, while effects are applied to the scope(s) following it.

We connect these transition systems through the relation *rdd*: *S* → *R*, where

$$\begin{array}{cc} \left(\forall s_{n}\in S\right) & \left(\forall a\in A\right)\left(\forall s_{m}\in S\right):s_{m}\in \gamma \left(s_{n},a\right)\;\Rightarrow \;\\ \left(\exists t\in T\right) & \left(\exists r_{n}\in R\right)\left(\exists r_{m}\in R\right):\\ & r_{n}=rdd\left(s_{n}\right)\\ & \wedge r_{m}=rdd\left(s_{m}\right)\\ & \wedge r_{m}\in \overset{≃}{\gamma }\left(r_{n},t\right)\\ & \wedge s_{m}\text{ satisfies }effects\left(r_{n}\right)\cup precond\left(r_{m}\right) \end{array}$$ (10)

**FIGURE 3:**
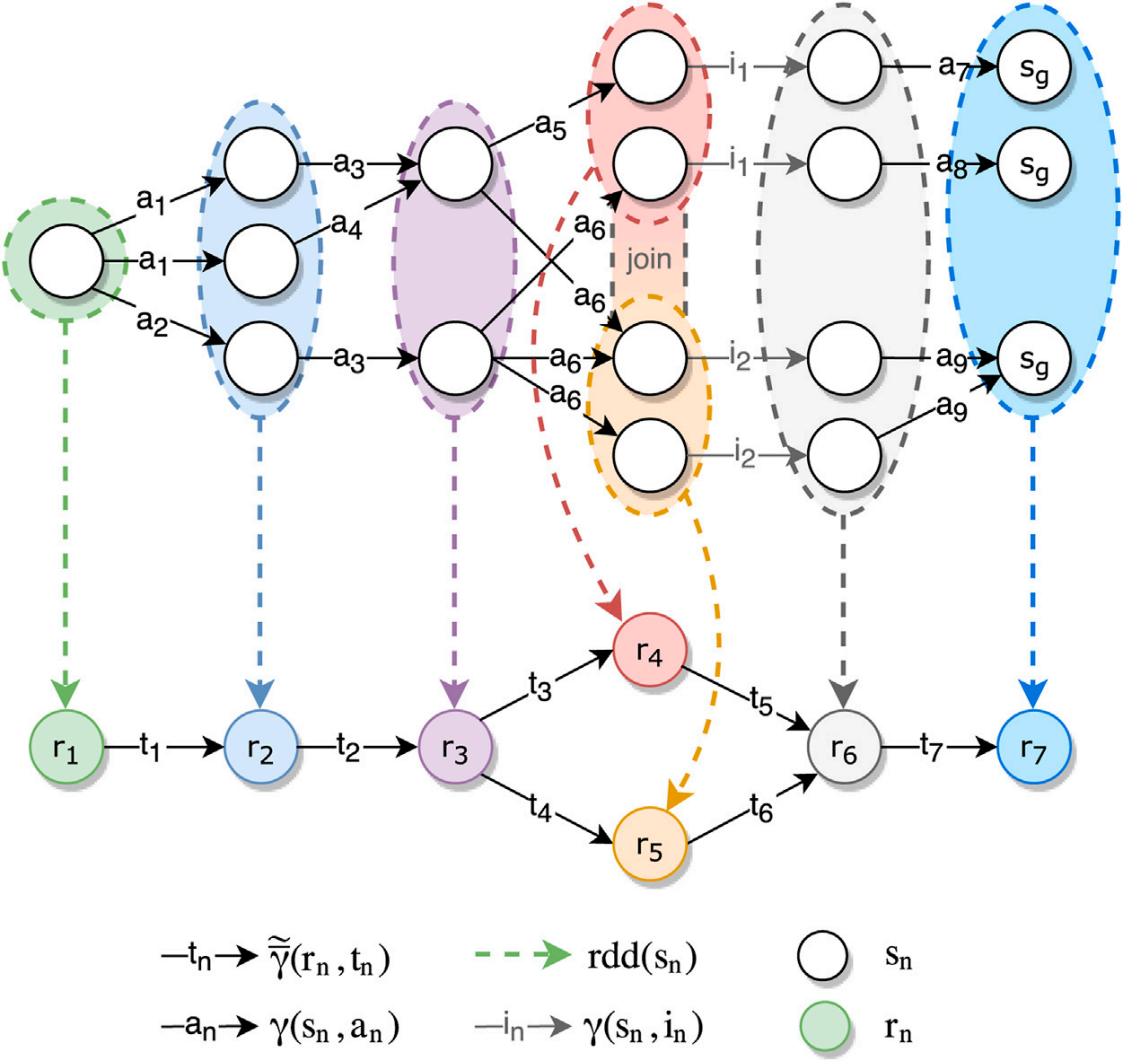
Example mapping between a Spark DAG $\overset{≃}{\Sigma }$ **(bottom)** and STS Σ **(top)**.

Figure 3 shows an example mapping between planning transition system Σ and a Spark DAG $\overset{≃}{\Sigma }$ , based on the scenario from Section 1. In this example, *γ* for some states contains multiple applicable actions (e.g., *a*_1_ and *a*_2_ can both be applied from the first state) or multiple possible result states based on an action (e.g., *a*_1_ from the first state leads to two next states if an *or-constraint* was used).

Since we only support planning for our variation points, we do not include in our planning problem any scopes that do not contain a variation point. On the other hand, we add a scope at the end of the pipeline so that we can attach the goal constraints, even if the final action of the pipeline does not contain a variation point.

We also add special handling for join operations. We show this in Figure 3 as a transition function in the form of *γ*(*s*_*n*_, *i*_*n*_). These transitions are applied when there is a transition in the Spark DAG that the user has no control over. For example, when joining two RDDs, the user cannot provide their own function that will be applied during the join. Nevertheless, we want to encode these transitions in order to accurately represent the pipeline. We introduce these *implicit transitions* in Section 4.2.3.

We use the relation *vp*: *T* → *VP* to map our transitions to variation points. This allows us to take into account additional constraints relating to the topology, such as the input/output combinations from Table 2, which we will discuss in Section 4.2.1. Additionally, we encode a constraint that ensures that if a single variation point is used in multiple transitions in a pipeline, the actions applied in those transitions are the same. This is done because one variation point can only hold a single PlanningModule and therefore can only be assigned a single action.

$$\begin{array}{cccc} \left(\forall s_{n},s_{m},s_{k},s_{l}\in S\right)\left(\forall a_{i},a_{j}\in A\right)\left(\exists t_{i},t_{j}\in T\right): & s_{m}\in \gamma \left(s_{n},a_{i}\right)\\ \wedge & s_{l}\in \gamma \left(s_{k},a_{j}\right) & \\ \wedge \; & rdd\left(s_{m}\right)\in \overset{≃}{\gamma }\left(rdd\left(s_{n}\right),t_{i}\right) & \\ \wedge \; & rdd\left(s_{l}\right)\in \overset{≃}{\gamma }\left(rdd\left(s_{k}\right),t_{j}\right) & \\ \wedge \; & vp\left(t_{i}\right)=vp\left(t_{j}\right) & & \;\Rightarrow \;a_{i}=a_{j} \end{array}$$ (11)

**TABLE 2:** Operation shape categories, where T and U are placeholders for some type and (T, U) represents a tuple of type T and U.

RDD		Function		Example operation	Compatible categories
Pre	Post	In	Out		
T	U	T	U	map	OneToOne
					OneToPair
					PairToOne
					PairToPair
T	T	T	Boolean	filter	OneToOne^a^
					PairToOne^a^
T	T	(T, T)	T	reduce	PairToOne
					PairToPair^b^
(T,U)	(T,U)	(U, U)	U	reduceByKey	PairToOne
					PairToPair^c^

a If action output is of type *Boolean*.

b If T is also a tuple.

c If U is also a tuple.

Expanding our definition of the transition function $s_{n}\overset{a}{\to }s_{m}$ with the isomorphism requirement, we get

$$\begin{array}{cc} s_{n}\overset{a}{\to }s_{m}\;\Leftrightarrow \; & s_{n}\text{ satisfies }precond\left(a\right)\\ \wedge \; & s_{m}\text{ satisfies }effects\left(a\right)\\ \wedge \; & \left(\forall v\in Vars\right):val\left(s_{n},v\right)=val\left(s_{m},v\right)\\ & \;\;\vee vaffectedByAction\left(a\right)\\ \wedge \; & \left(\exists t\in T\right):rdd\left(s_{m}\right)\in \overset{≃}{\gamma }\left(rdd\left(s_{n}\right),t\right)\\ \wedge \; & s_{m}\text{ satisfies }effects\left(rdd\left(s_{n}\right)\right)\\ \wedge \; & s_{m}\text{ satisfies }precond\left(rdd\left(s_{m}\right)\right) \end{array}$$ (12)

#### 4.2.1 Data and Operation Types

An important property of Apache Spark pipelines and of most distributed data processing pipelines in general is typed data. Each vertex in the DAG has a type, and operations can change the data type. We, therefore, add *Type* as a variable to each planning state, and actions can change this type from one state to the next. In order to properly represent the intricacies of a Spark pipeline, we treat the *Type* variable differently from other state variables. In the first place, this is because we can infer the appropriate type constraints from the provided actions and pipeline topology. A second reason is that Spark treats RDDs differently depending on if they contain a single value or a tuple of two values (a value pair). For example, some operations (such as reduceByKey) require an RDD with a value pair to be applicable, while for others, it does not matter. Furthermore, some operations (such as map and reduce) can transform between these categories, while others require the input/output multiplicity to be the same (such as filter). In our planner, we must therefore distinguish between these input/output categories, labeled OneToOne, OneToPair, PairToPair, and PairToOne. We store this category in the variation point and then ensure only appropriate actions are selected, with “categoryOf” as a lookup for the category:

$$\begin{array}{cc} s_{n}\overset{a}{\to }s_{m}\;\Rightarrow \; & \left(\exists t\in T\right):\\ & rdd\left(s_{m}\right)\in \overset{≃}{\gamma }\left(rdd\left(s_{n}\right),t\right)\\ & \wedge \;|\;vp\left(t\right)|=1\\ & \wedge \text{ categoryOf }vp\left(t\right)=\text{ categoryOf }a \end{array}$$ (13)

Apart from the RDD types, the applicability of an action is also dependent on the logic of the operation itself. For example, consider an RDD of type T. When you apply a plannedFilter operation, the filter function receives the same type T as input but gives a Boolean as output. However, Spark uses that result to filter the dataset and returns an RDD that still contains the same T type. The different combinations used by Spark are listed in Table 2.

To be able to handle all required type restrictions, we have split our *Type* variable into a *TypeLeft* and a *TypeRight* variable, representing the left and right elements of a tuple, respectively. For types that are not tuples, only the value for *TypeLeft* will be set, and *TypeRight* will be set to a null value. This allows us to specify constraints based on only one part of the data type, for example, with *TypeLeft* = *Boolean* for plannedFilter.

To distinguish between the RDD type and Function type from Table 2, type information is encoded differently depending on the Spark operation used. For example, for plannedReduceByKey with a PairToOne module, we encode our constraints as

$$\begin{array}{cc} s_{n}\overset{a}{\to }s_{m}\;\Rightarrow \;val\left(s_{1},TypeLeft\right) & =val\left(s_{2},TypeLeft\right)\\ \wedge val\left(s_{1},TypeRight\right) & =val\left(s_{2},TypeRight\right)\\ & =inLeft\left(a\right)\\ & =inRight\left(a\right)\\ & =outLeft\left(a\right) \end{array}$$ (14)

with *inLeft*(*a*) and *inRight*(*a*) describing the input types for action *a* and *outLeft*(*a*) describing the output type for *a*. Since *a* represents a PairToOne module, *outRight*(*a*) would return ∅.

#### 4.2.2 No-Operation

Some pipeline topologies may contain more operations than required to fulfill the planning goal. We introduce the no-op action *τ* (De Nicola and Vaandrager, 1990; Ghallab et al., 2004) so that we can still assign an action to every variation point in the pipeline, while not performing unnecessary computations. We create both a NoOpOneToOne and a NoOpPairToPair action but do not include no-op actions for the OneToPair and PairToOne categories as performing any operation on them would not be idempotent.

#### 4.2.3 Join States

Finally, we must support pipelines that contain join operations such as the union of two datasets. To simplify our planner, we restrict our implementation to join operations that do not change the datatype. We initially support the union and intersection operations.

**FIGURE 4:**
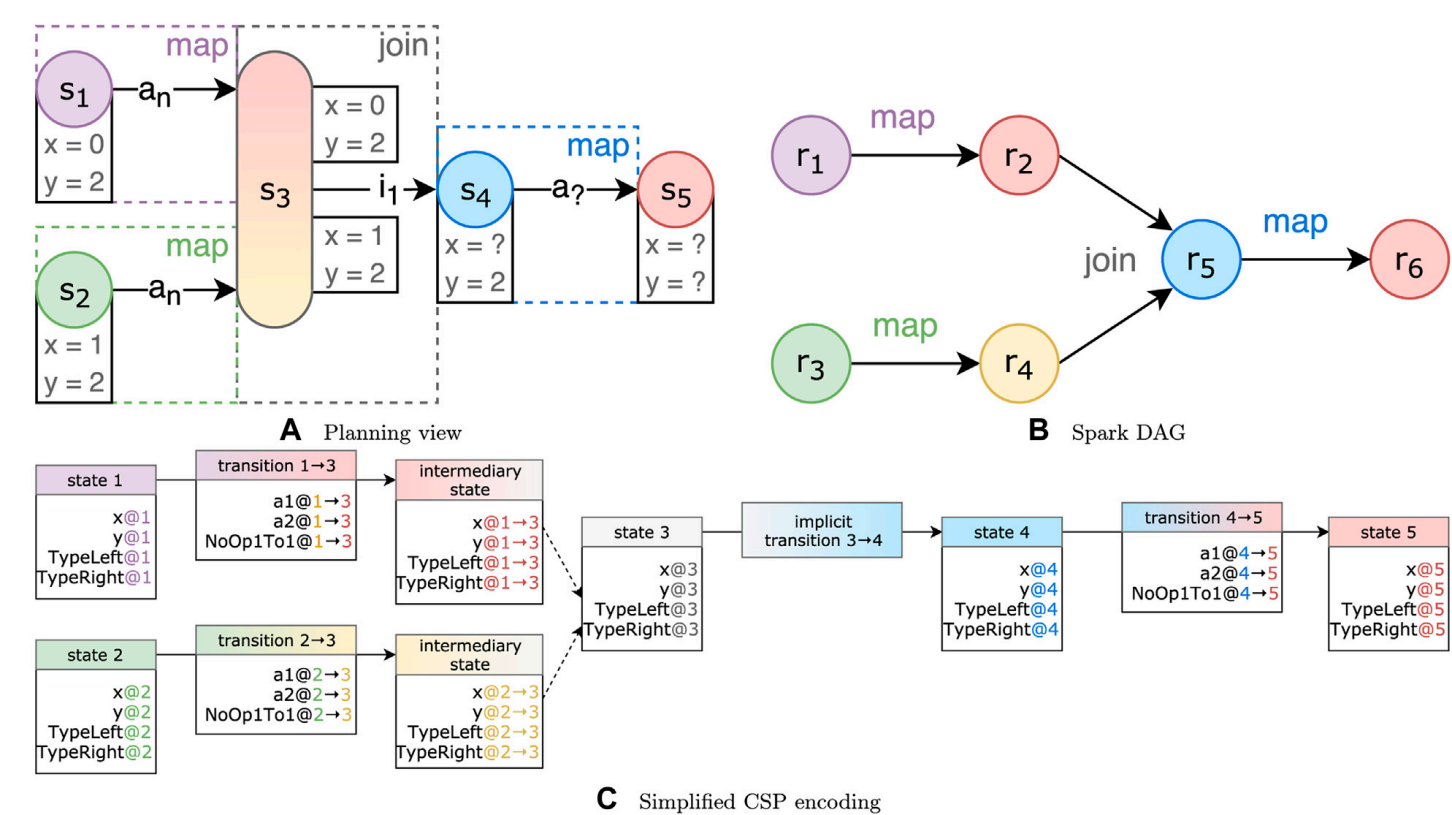
Three views of an example transition system with a join state and conflicting state variables. Variable *x* in *s*_4_ could have multiple values.

To illustrate why we need to explicitly add support for join operations, consider the example illustrated in Figure 4. Here, two states with conflicting state variables are joined. For variable *y*, it is clear that, in *s*_4_, the state variable *y* = 2. However, for variable *x*, it could be true that either *x* = 0 or *x* = 1.

There are two strategies to resolve this conflict:

• Require equality
  If we add a constraint requiring both values to be equal [e.g., *val*(*s*_3_, *v*) = *val*(*s*_1_, *v*) = *val*(*s*_2_, *v*)], there will be no uncertain values in *s*_4_. The actions applied onto *s*_1_ or *s*_2_ will have to be changed so that all state variables end up with the same values, in order to generate a valid plan.
• Accept either
  By accepting either value, we say *val*(*s*_3_, *v*) = *val*(*s*_1_, *v*) ∨ *val*(*s*_3_, *v*) = *val*(*s*_2_, *v*). This introduces nondeterministic behavior in the steps following *s*_3_. We do not apply this functionality to the *Type* variable, as the RDD resulting from the join must always be set to a single data type.

The “require equality” strategy can be too rigid for certain pipelines, while the “accept either” strategy can greatly enlarge the search space of our planner since there are more possibilities to try. We, therefore, allow the user to set the resolution strategy for each join individually.

Since join operations do not allow custom code, we should not allow the selection of any action for these operations. We, therefore, modify the STS to include *implicit transitions* that reach the next state without an action being applied. We could redefine *γ* to support this but for simplicity, we instead define an implicit transition as a regular transition that always only applies the no-op action.

We also add *intermediary states* on which constraints of the transitions before the join states are encoded. If we directly encode the constraints onto the join state, both sets of constraints must always hold, and the “accept either” strategy would not be respected. Instead, the intermediary states allow us to evaluate the strategy over the intermediate variables of each intermediary state, represented as the white boxes in Figure 4A.

### 4.3 Planner Representation as CSP

Now that we have a formal description of our planning model; we can describe how we have implemented it. We have used the Java-based Choco-solver (Prud’homme et al., 2017) library to implement our CSP-based planner, which has good interoperability with Scala.

We encode each “state variable” as a CSP variable [e.g., *val*(*s*_1_, *y*) is encoded as variable *y@*1]. The domain of the variable is the set of possible assignments found in the planning problem. All values are converted to integers in the CSP. For the *Type* variables, we first convert a type name into a fully qualified name [e.g., Seq(String) becomes “scala.collection.Seq(java.lang.String)”], and for each unique name, we assign a unique integer in the domain. The actions applied on transitions are tracked through “action variables” (e.g., *a*1*@*1 → 3), where a value of *true* indicates that the action (in this case *a*1) is applied in that transition (in this case 1 → 3). Figure 4C shows a CSP encoding of the join example discussed in subsection 4.2.3.

Encoding the constraints from Eq. 5 defined on the topology and on actions is also straightforward, since equality and inequality can be encoded as constraints {e.g., *val*(*s*, *v*) = *b* becomes arithm[v@s, “=,” encode(b)]}, with encode being the conversion of values described above. Conjunction and disjunction of constraints can also be encoded in the CSP [e.g., *val*(*s*, *v*_1_) ≠ *val*(*s*, *v*_2_) becomes arithm(v1@s, “!=” v2@s)].

By only creating transitions between the scopes following the Spark DAG, we automatically fulfill part of the requirement of the plan to be isomorphic to $\overset{≃}{\Sigma }$ such that $s_{n}\overset{a}{\to }s_{m}\;\Rightarrow \;\left(\exists t\in T\right):rdd\left(s_{m}\right)\in \overset{≃}{\gamma }\left(rdd\left(s_{n}\right),t\right)$ .

Subsequently, we add a constraint stating that exactly one of the action variables in each transition can be used. If we treat a false Boolean value as zero and a true Boolean value as one, we can add a constraint that sums each action variable in a transition and ensures the sum is equal to one.

Finally, we add an optimization objective that maximizes the occurrences of the no-op actions, to ensure that we do not perform extremely unnecessary processing (e.g., performing some function *f*, undoing it, and then redoing it).

From the action variables defined on the transitions in the CSP, we can then extract the actions assigned on those transitions, which correspond to user code that should be assigned to variation points.

### 4.4 Planning Model Justification

Having defined our planning system, we must first know that it provides correct results before it can be used in practice. This is based on the concepts of *soundness* and *completeness* (Ghallab et al., 2016). A planning system is sound if, for any solution plan it returns, the plan is a solution for the planning problem. A system is complete if, given a solvable planning problem, the system will return at least one solution plan.

Because we represent our planning problem as a CSP and use an existing constraint solver, we do not evaluate the planning problems ourselves. Nevertheless, we can guarantee that the planning process is *complete*; i.e., it will eventually stop and result in either a generated configuration or a failure. This is true because our planning problem is finite (the Spark DAG is of finite size, each state in Σ has a finite number of state variables, and each state variable has a finite domain) and as a result, the encoded CSP also contains a finite number of variables with a finite number of domains. Since the CSP solver works through piecemeal reduction of the variable domains, given a correct solving algorithm (Kondrak and van Beek, 1997), eventually, a solution will be reached or the solving will fail in case the CSP is unsatisfiable. The result is also *sound* since we encode all aspects of our planning problem as constraints as described in the previous section (i.e., every possible variable and its type and all possible actions), and the constraint solver will ensure every constraint on the CSP is met. Therefore, any solution to the CSP is a solution to the planning problem.

Soundness and completeness of the planning problem itself are based on both the construction of the transition system Σ as well as the representation of the distributed processing pipeline $\overset{≃}{\Sigma }$ . This is again focused on the Spark DAG but can be modified for other distributed processing frameworks. Since our planning system is based on the state-variable representation as described by Ghallab et al. (2004), we know that this approach can yield correct results. Therefore, we will only discuss the correctness of the Spark DAG translation, i.e., $\overset{≃}{\Sigma }$ . We do this based on our definitions in Section 4.2, of which the most important is the final definition of the transition function in Eq. 12.

Since Spark uses a (directed) acyclic graph, the transition system must also be acyclic. We represent the DAG as $\overset{≃}{\gamma }:R\times T\to R$ . Since $\overset{≃}{\gamma }$ is created from the Spark DAG, these properties (such as it being acyclic) also hold for $\overset{≃}{\gamma }$ . Next, recall the *rdd* relation between $\overset{≃}{\gamma }$ and *γ*, defined as $\left(\forall s_{n}\in S\right)\left(\forall a\in A\right)\left(\forall s_{m}\in S\right)\left(\exists t_{i}\in T\right):s_{m}\in \gamma \left(s_{n},a\right)\;\Rightarrow \;rdd\left(s_{m}\right)\in \overset{≃}{\gamma }(rdd\left(s_{n}\right),t_{i}).\text{If there}$ If there isa cycle between states in *γ*, there must then also be a cycle between RDD scopes in $\overset{≃}{\gamma }$ . This is not possible; therefore, *γ* must also be acyclic.

Apart from relating *γ* with $\overset{≃}{\gamma }$ as described above, the *rdd* relation also ensures that topology constraints that specify the behavior of the pipeline are met. This is achieved through the encoding of *effects*(*rdd*(*s*_*n*_)) and *precond*(*rdd*(*s*_*m*_)) as constraints that must be satisfied, given (*∃t* ∈ *T*) such that $rdd\left(s_{m}\right)\in \overset{≃}{\gamma }\left(rdd\left(s_{n}\right),t\right)$ . If there is no transition *t*, that means there is no *s*_*n*_ ∈ *γ* that results in *s*_*m*_. In this case, *s*_*m*_ is an initial state and as such, it is already described by the initial conditions *S*_0_.

Further complications arise from the possibility of multiple RDDs to be joined, for which our solution is described in Section 4.2.3. We support two strategies that reconcile the state variables between the two branches. For the “require equality” strategy, all state variables are related through an equality constraint. If a state variable in one branch is different from the other, this constraint would be violated and the configuration would not be provided as a solution. For the “accept either” strategy, the user explicitly states that such conflicting state variables are still acceptable in a solution for the planning problem. Nevertheless, the final Spark configuration is still limited in that an RDD can only be assigned a single type. As a result, a solution where multiple possible types are assigned to a single state could not be applied to the pipeline. As mentioned in the description of the “accept either” strategy, we slightly restrict the planning model by specifying that the *Type* variables should always be equal between branches regardless of the chosen strategy.

Another complication of join operations is that they are the only operations supported by our planning system that do not accept a user function (in which an action could be applied). A configuration would therefore be invalid if an action is assigned to such an operation. Through the implicit transitions mentioned in Section 4.2.3, we enforce a constraint that ensures a no-op action is applied in those transitions; therefore, no invalid action can be assigned.

Finally, through the *vp* relation, we ensure that a module is of the same category as the variation point in an operation. Furthermore, we ensure that the module is compatible with the operation. If the user attempts to use a variation point in an operation of a type that is not compatible, the chosen module must be of the same incompatible type. As this is prevented in our action encoding, no plan can be generated.

## 5 Evaluation

In this chapter, we evaluate our planning and runtime updating system with respect to its runtime performance. We measure the performance using three different experiments:

• Plan generation time
  Determine how long it takes to generate a plan for a pipeline with either a varying number of steps, a varying number of possible actions per step, or varying numbers of joins.
• Runtime overhead
  Determine the difference in performance between our solution and regular Spark, excluding the time it takes to perform the configuration planning process.
• Restarting experiment
  Quantify the performance of reconfiguring the pipeline by updating running scenarios versus having to restart the entire application.

We performed experiments several times for a fair and stable evaluation. Each experiment has been run a total of six times, each time executing in the above order.

The experiments were run on a dedicated cluster consisting of one master and five slaves, running in Spark’s standalone cluster mode. All six machines have the same specifications, listed in Table 3.

**TABLE 3:** Experiment cluster node specifications.

Spark version	2.1.0
Java version	jre-1.8.0-openjdk
Operating system	CentOS 6.8 64 bits
Processor	2.7 GHz AMD hexacore
Memory	48 GB, 1,333 MHz DDR3


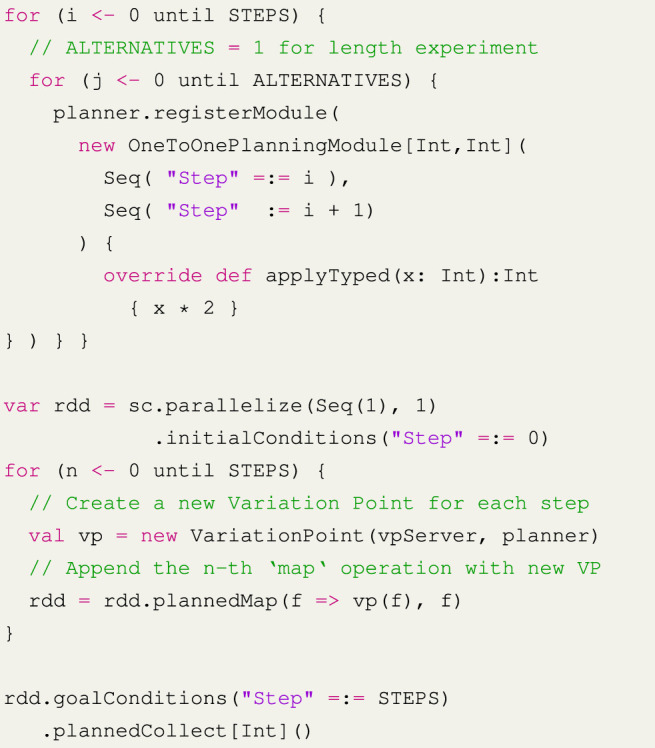


### 5.1 Plan Generation Time

The plan generation time experiments explore three different properties of a pipeline to determine their impact on the time it takes to generate a configuration.

**FIGURE 5:**
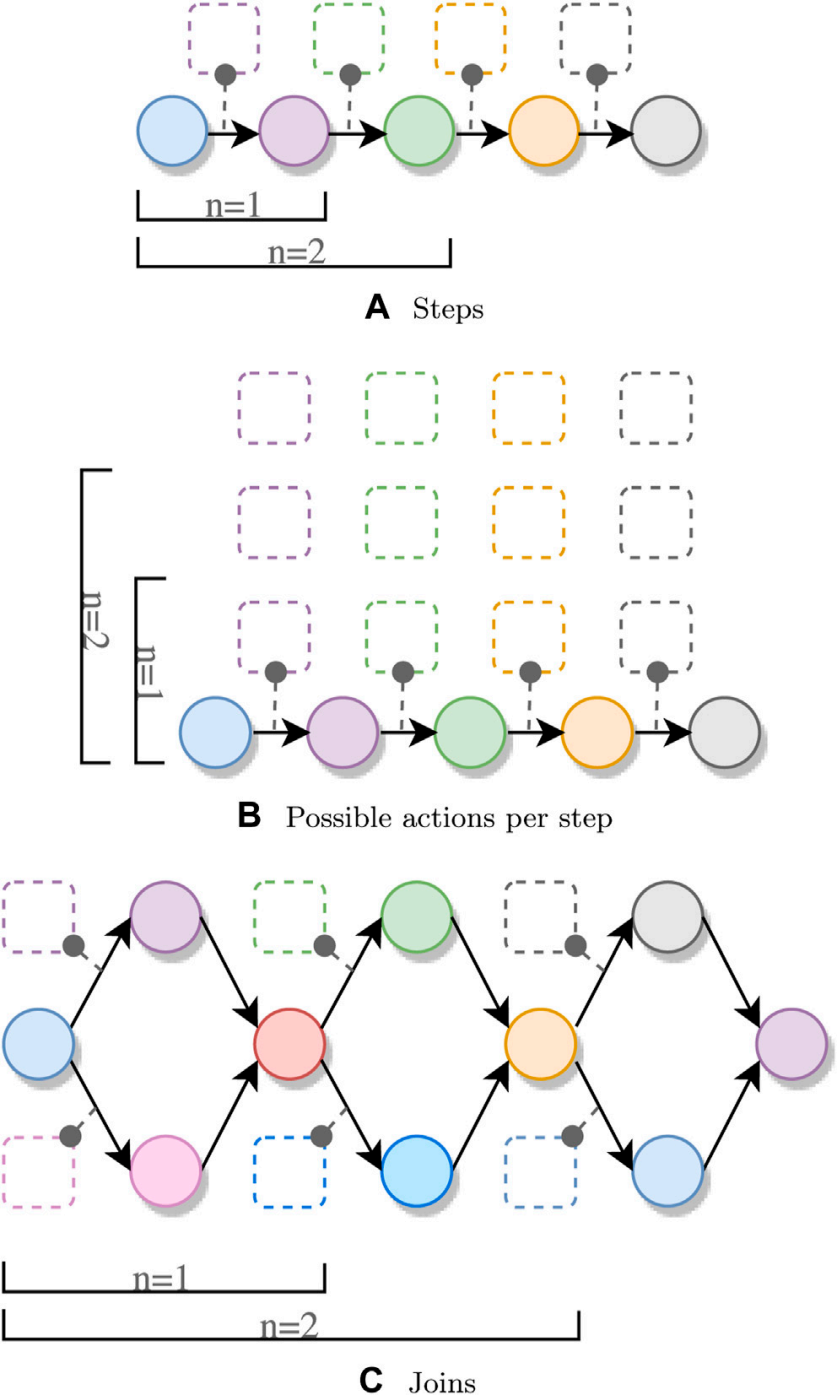
Plan generation for a varying number of **(A)** steps, **(B)** possible actions per step, and **(C)** joins. Circles and rectangles represent steps and actions, respectively.

The first property we examine is pipeline length. We measure how long it takes to generate a plan for a pipeline with *n* sequential operations, as shown in Figure 5A. We first measure the planning time for only one operation, followed by the planning time for two operations, up until *n* = 16. For each step, only a single action is applicable. This is achieved by generating an action specifically for each step in the pipeline, as shown in Listing 1.

Listing 1 Overview of the generator for variable length pipelines.

The second property being examined is the number of alternatives per step, as shown in Figure 5B. We generate a pipeline with four steps, but instead of having exactly one action for each Spark operation, we generate multiple actions with the same preconditions and effects on the *Step* variable, as shown in Listing 1 with ALTERNATIVES > 1.

The last property under examination is the number of joins in a pipeline. We again allow only one possible module per step, but instead of applying a single operation to the initial RDD, we apply two separate map operations on the same RDD, followed by a union. The resulting pipeline topology is shown in Figure 5C. We determine the time it takes to generate such pipelines for both join strategies mentioned in Section 4.2.3: “require equality” and “accept either.” We otherwise generate the pipeline in the same way as Listing 1.

**FIGURE 6:**
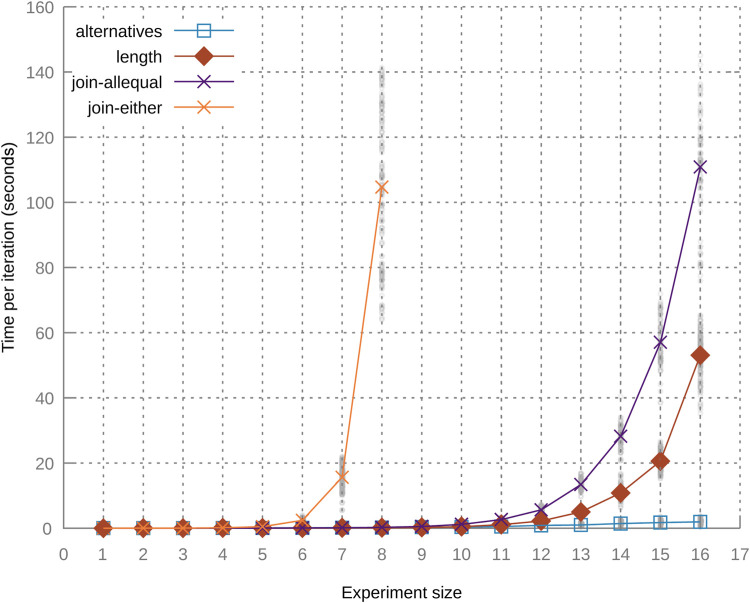
Results of the plan generation time experiments. The lines represent the average value of each measured property, and dots represent individual measurements.

The results of all measurements in this experiment are shown in Figure 6.

Let us first discuss the results for the varying number of operations and number of alternative actions per operation. For both measurements, the growth is exponential, although the planning generation time for the variable pipeline length grows considerably faster.

We attribute this higher growth rate to multiple factors. First, the domain for the *Step* variable increases, as the final goal value is increased. Next, the system also needs to test the applicability of more possible actions, since we generate one action for each step in the pipeline. For the steps themselves, we need to encode more constraints (described in Section 4), and we also have more steps where we try to apply the no-op actions. The measurements for the varying number of alternatives are also affected by the increase of possible actions to be tested; however, this introduces much fewer constraints to the CSP.

The results for the experiments using a variable number of joins show a higher growth rate when using the “accept either” strategy compared to the “all equal” strategy. The planning time when using the “all equal” strategy still grows faster than that of the varying pipeline size and varying number of alternatives, since for each join, two actions need to be applied, one for each branch. Using the “accept either” strategy results in even bigger planning time growth, since the system must accept either value as a result of one join. This uncertainty propagates through every step in the pipeline, meaning that we cannot reduce the search space as quickly as for the other experiments.

### 5.2 Dynamic Versus Static

Next, we compare the adaptive framework with the *spark-dynamic* framework (Lazovik et al., 2017) and with regular Spark implementations. These experiments are based on three implemented scenarios inspired by real projects, each built using commonly used operations and increasing in complexity.

#### 5.2.1 Scenarios

**FIGURE 7:**
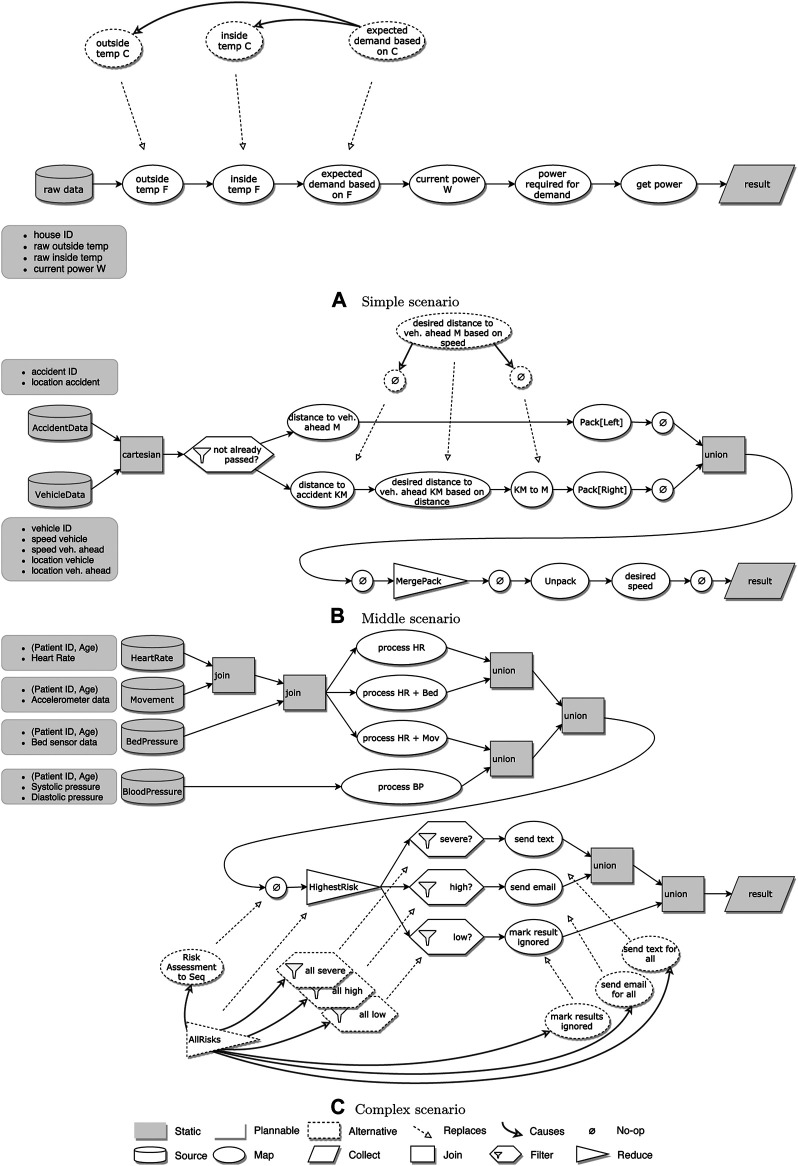
Pipelines for three different scenarios: **(A)** simple, **(B)** middle, and **(C)** complex.

The simple scenario is from the Energy domain. Based on the temperature inside and outside a house, we calculate the power required to heat the house based on an ideal temperature. Accurately estimating the future power usage of a building may allow more efficient distribution of available power within Smart Buildings (Georgievski et al., 2012) or Smart Power Grids (Kok, 2013), resulting in lower energy costs. A representation of this pipeline is shown in Figure 7A.

The middle scenario is based on autonomous driving. In this scenario vehicles are driving along a highway that has been outfitted with sensors that track the location of all vehicles driving on it, as well as the location of any accidents that happen on that highway. We focus our scenario not on autonomous driving itself, but on a small part of the information processing. When a vehicle approaches an accident, laws or safety requirements could indicate that a vehicle should have a specific (minimum) distance to the vehicle in front of it. In this scenario, we calculate the speed required to reach the required distance based on several factors: the location of the accident (from the starting point of the roadway), the speed and location of the current vehicle, and the speed and location of the vehicle ahead. We use this data to calculate the distance from the current vehicle to the vehicle ahead and the distance from the current vehicle to the accident. The pipeline used in this scenario is shown in Figure 7B. This scenario is a bit more complex than the previous one because the pipeline contains a split and join as well as operations other than just plannedMap.

The final scenario is the most complex, containing multiple splits and joins as well as multiple types of operations. This scenario is related to the healthcare domain. In the scenario, patients are being monitored remotely based on their heart rate, blood pressure, how much they are moving, and whether they are in their bed. Several risk assessments are done relating to the health of the patients based on this data. For example, if the heart rate of the patient is high and they are not moving, something might be wrong. By creating a plannable pipeline for this monitoring, changes can be made without having to temporarily interrupt the monitoring process, which could result in dangerous or relevant medical situations being missed. The pipeline for this scenario is shown in Figure 7C.

Each of these scenarios is implemented in three different ways:

• Static
  This implementation is a regular Spark pipeline as it would be written without the system described in this paper.
• Dynamic
  The dynamic implementation uses the variation points from *spark-dynamic* for each operation, with the variation points given preassigned functions.
• Planned
  The planned version uses the planner and plannable variation points from this paper.

The planned implementations of the simple and complex scenarios also include alternative PlannedModules that can fulfill the scenario goals. The middle scenario instead contains extra variation points that should be assigned no-op actions. This way, all three scenarios are given a bigger search space during the planning process. For the static implementation of each scenario, only the base scenario is implemented, since updating it is not possible. The dynamic implementations also only contain the base scenario. This is because the Spark pipeline is still restricted to static RDD types with the *spark-dynamic* library. The implementations of the scenarios can be found in an external repository^2^. This includes the code used to generate the input data. We have also added detailed DAG representations to the repository for each scenario that includes pipeline constraints and assigned PlannedModules.


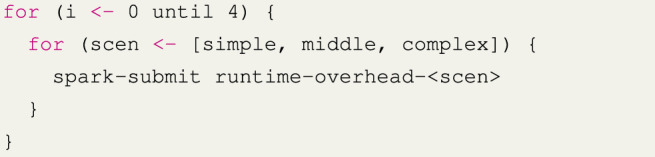



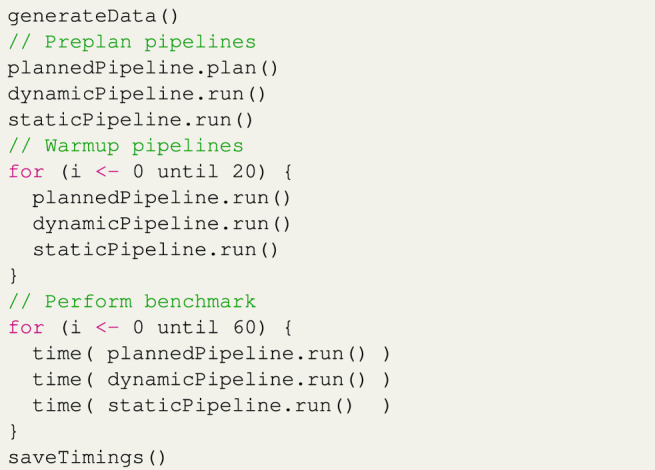


#### 5.2.2 Runtime Overhead

In the runtime overhead experiment, we run each implementation of every scenario and measure how long one iteration takes to complete. For the planning implementation, we do not replan the pipeline between iterations, as we are just interested in the actual runtime overhead. Within this experiment, all scenarios (simple, middle, and complex) were executed 240 times. Every 60 iterations, the application terminates so that any optimization to the bytecode done by the JVM during a run does not greatly affect the benchmark. This scheduling is shown in Listing 2. Since each experiment is repeated six times, each scenario is started four times and each scenario runs the pipeline 60 times; in total, we have 6 × 4 × 60 = 1440 measured runs.

Listing 2 Scheduling of the runtime overhead experiment.

Each iteration of the simple scenario is run with 4,400 input objects, the middle scenario is run with 1551 × 2 input objects from two source RDDs, and the complex scenario is run with 6600 × 4 = 26400 input objects from four source RDDs.

Listing 3 shows how a single run of this experiment is performed. First, we randomly generate the data that will be used for that run of the experiment. Next, we run the pipeline once and store the generated plan since that is not a factor we want to test with this experiment. We then perform warmup cycles on the data to eliminate JVM startup interference, followed by timing the real benchmark cycles.

Listing 3 Overview of the runtime overhead experiment code.

**FIGURE 8:**
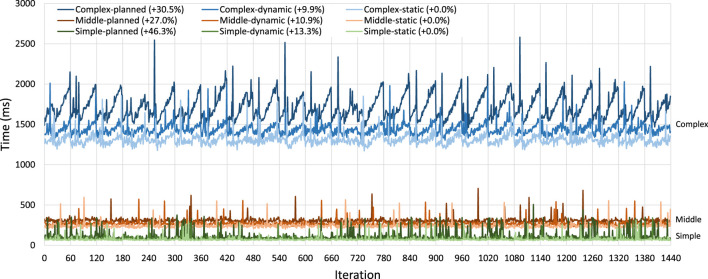
Results of the runtime overhead experiment per scenario. Each scenario is given a color, with the lightest, the mild, and darkest shades of each color representing the *static*, the *dynamic*, and the *planned* implementations, respectively. The vertical lines indicate 60 pipeline iterations, after which the Spark application is restarted.

The results of this experiment are shown in Figure 8, Table 4. First, the *dynamic* implementation of each scenario takes longer to run than the *static* Spark implementation. This matches the results of the earlier experiments done for *spark-dynamic* (Lazovik et al., 2017). The reason for this is that we have added extra functionality on top of the existing static Spark code.

Looking at the results for the simple scenario, when using the *static* implementation as a baseline, the *dynamic* implementation takes approximately 13*%* longer since we have to download the assigned contents of the variation points and process them. The running time of the *planned* implementation is approximately 46*%* longer than the baseline. This is because we do not only have the overhead of the *dynamic* implementation but also have extra logic to enable the dynamic typing, such as casting the input data. The loading of variation points is also slightly more complicated since the library must make sure that planning has been completed. Individually, these steps would not take much time but since this is repeated for every record of every operation in the pipeline, their effects become significant. The *planned* implementation also suffers (more than the other implementations) from irregular increases in the time per iteration, which could be the result of networking lag, thread scheduling, or garbage collection.

The results for the middle scenario are similar, with the *dynamic* implementation having an overhead of approximately 11*%* above baseline, and the *planned* implementation having an overhead of 27*%*. Since the average iteration time for this scenario is longer than the simple scenario, the irregular spikes mentioned above have a smaller effect on the averages.

The results for the complex scenario show overhead for the *dynamic* implementation of approximately 10*%* above baseline and overhead of approximately 31*%* over the *static* baseline for the *planned* implementation. These results show a new phenomenon, where the iterations appear to increase in running time every iteration until the Spark application is restarted. This is primarily the result of garbage collection performed by the JVM. Switching to a different garbage collection implementation or changing the memory size allocated to Spark executors changes the curves of the results.

**TABLE 4:** Averaged results of the runtime overhead experiment per scenario.

Impl.	Scenario					
	Simple (ms)		Middle (ms)		Complex (ms)	
Static	76.00	—	241.62	—	1311.58	—
Dynamic	86.08	(+13.3*%*)	267.85	(+10.9*%*)	1441.57	(+9.9*%*)
Planned	111.18	(+46.3*%*)	306.85	(+27.0*%*)	1711.74	(+30.5*%*)

#### 5.2.3 Restarting Experiment

In this experiment, we determine how much increase in performance we can achieve by using the planning system. We define the performance gain as the difference in time it takes to process a set of data while performing reconfiguration at runtime compared to having to restart a static Spark application.

**FIGURE 9:**
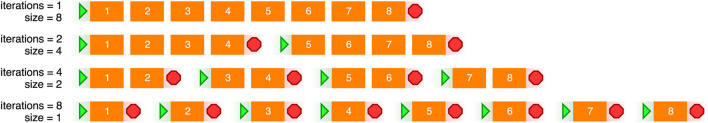
Overview of the restarting experiment, with the start and stop symbols representing the reconfiguration of the pipelines.

A basic overview of this experiment is shown in Figure 9. Here, we split the dataset into sections and first process all eight sections of the dataset. For the *static* implementation, this means we only start the application once, and for the *planned* implementation, this means we only generate a new plan once. In the next test, we only process half of the dataset before a reconfiguration takes place: for the *static* implementation, Spark terminates after each section of the dataset and we restart it for the next dataset; for the *dynamic* case, we simply start the next iteration without making any changes; for the *planned* case, our system must generate a new plan. After the reconfiguration, we continue processing the other half. We continue subdividing the dataset for these tests until we have to reconfigure the application after every section of the dataset.

In the actual experiment, we do not use just eight sections of the data as described above but instead use the scheme shown in Listing 4, e.g., one iteration with 80 copies of the database, followed by two iterations of 40 copies of the database, etcetera. During experimentation, the number of iterations/slices per dataset was increased until a stable trend was found at 20 iterations per dataset and further increased to 80 iterations to ensure that the trend remained stable. Since the *static* implementation cannot be updated, it is fully restarted for every iteration. Listing 5 shows a rough overview of the applications used in the experiment.

Listing 4 Scheduling of the restarting experiment.

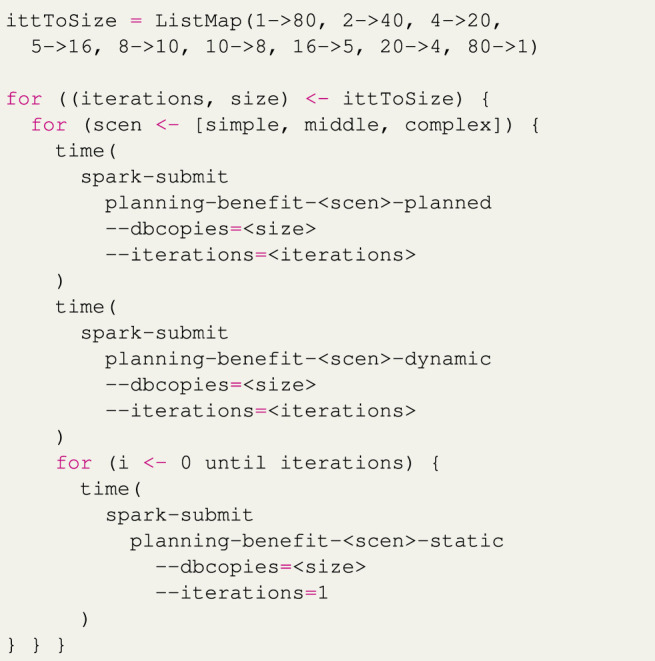

Listing 5 Overview of the restarting experiment code.

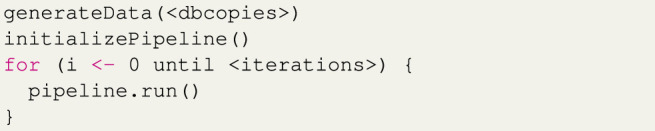

**FIGURE 10:**
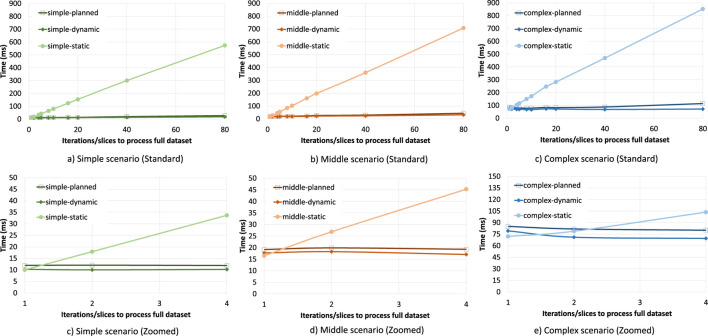
Results of the restarting experiment per scenario. The zoomed subfigures show the breakdown for each scenario. Each scenario is given a color, with the lightest, the mild, and darkest shades of each color representing the *static*, the *dynamic*, and the *planned* implementations, respectively. *Iterations to process full dataset* indicates the number of sections in the dataset. With one iteration, the entire dataset is processed without restarting, while with 80 iterations, the pipeline is restarted 79 times to process all data.

The averaged results of these experiments are shown in Figure 10. We see that if the *static* implementations have to be restarted 80 times, it takes a considerable amount of time for all scenarios. Restarting only 40 times takes around half as much time. The *dynamic* implementations take a roughly constant amount of time for each scenario, as there is barely any cost apart from the actual processing of the sections of the datasets. The *planned* implementations show a slight linear increase in processing time when the number of reconfigurations is increased, and this is most pronounced for the complex scenario. This increase is the result of the planning process, where a plan has to be generated and the variation points have to wait for the planning process to complete for each iteration. The *planned* implementations nevertheless perform much better in this experiment than the *static* implementations.

Table 5 shows the results of this experiment for the cases where the dataset is processed without restarting and where the dataset is divided into 80 sections. Similar to the previous experiment, when the dataset is processed without restarting, the *static* implementation of each scenario outperforms the other implementations. However, when the reconfiguration is done 80 times, the *planned* implementations process the entire dataset in around 90% less time than it takes for the *static* implementations to finish. The results when the dataset is processed without restarting only roughly match those of the runtime overhead experiment since that experiment does not include the planning and application startup time in its measurements and its RDDs contain much fewer records.

In this experiment, we assumed that the data is not dependent and can be split into chunks, and all updates are anticipated. However, in many cases, the same algorithm should be applied to the whole dataset where a restart will lose progress, and the updates are unforeseen. For example, a pipeline with only two steps, *α* and *β*, is running. While *α* is almost over, you will find a bug in *β*. Applying a bug fix in the static implementation means losing *α*’s computations while using our system *β* can be updated separately. In the worst-case scenario, the planned implementation introduced 46% overhead, which means even a single update after 46% of the pipeline runtime will benefit from planned implementation. Obviously, the planned implementation will outperform the static implementation in multiple update scenarios. It is noteworthy to mention that this is just a proof-of-concept implementation of our system.

**TABLE 5:** Results of the restarting experiment per scenario for 1 and 80 iterations.

Itt	Impl.	Scenario					
		Simple (s)		Middle (s)		Complex (s)	
1	static	9.6	—	16.8	—	71.6	—
	Dynamic	10.4	(+8.4*%*)	17.6	(+5.0*%*)	78.5	(+9.7*%*)
	planned	12.1	(+25.8*%*)	19.2	(+14.3*%*)	86.2	(+20.3*%*)
80	static	572.9	—	708.4	—	854.6	—
	dynamic	18.5	(−96.8*%*)	31.6	(−95.5*%*)	71.1	(−91.7*%*)
	planned	26.8	(−95.3*%*)	43.3	(−93.9*%*)	114.0	(−86.7*%*)

## 6 Conclusion and Discussion

In this work, we have introduced a system for adaptive on-the-fly changes in distributed data processing pipelines using constraint-based AI planning techniques. The feasibility of the approach is tested using Apache Spark as a target distributed processing framework. In this paper, we also present the generic methodology that enables adaptive on-the-fly changes of applications in distributed data analysis for industrial organizations in the Industry 4.0 era. While the proof-of-concept implementation is specific to Apache Spark, the methodology and planning model can be applied to any distributed data process platform operating on the same principles (that is, through a sequence of operations forming a DAG that allows custom user code to be executed). Regarding the proof of concept itself, rapid development and modification of running pipelines could in some cases already benefit from the use of this system.

The results of our experiments show the exponential nature of the planning time, which is dependent on the computational complexity of the planning problem itself (pipeline length, number of alternative actions, and number of joins). The results also show the overhead introduced by the additional functionality that enables dynamic typing, compared to the more restrictive *spark-dynamic* system (Lazovik et al., 2017).

We also note that our evaluation was performed using comparatively small datasets, with between 4,400 and 264,000 entries per RDD in the runtime overhead experiment. As a result, the overhead introduced by both *spark-dynamic* and our planning system could be overemphasized compared to real-world usage of the system. However, this did allow us to repeat the experiments multiple times.

We believe that the system described in this paper provides a solid foundation and starting point for automated DSU systems for distributed data processing frameworks, where the general feasibility of this approach is shown through our implemented scenarios and their evaluation.

Since this work is one of the first attempts at integrating adaptive reconfiguration and DSU to the field of distributed data processing, a lot of directions for possible future research exist. Due to the novel nature of this research, we do not consider this as a weak point but instead as an opportunity for further development of this field. Techniques such as distributed planning, optimized replanning (fewest changes compared to the previous plan), compile-time validation, and preplanning can increase the performance of the planning process, as well as pipeline development in general. Furthermore, allowing extended goals (such as achieve-and-maintain), partial knowledge, dynamic goals (updating topology constraints), and planning over multiple pipelines can increase the usefulness of the system. Another beneficial feature would be the ability to perform replanning for batch pipelines where some steps have already been completed, in which case only uncompleted steps should be replanned. Finally, implementing reconciliation strategies for in-transit data (when a pipeline is updated while processing) and allowing dynamic pipeline topologies are important points that still need to be addressed. It is also important to investigate in which cases a dynamic updating framework as described in this paper should (or should not) be used, which will require using it in operational settings in different domains.

## Data Availability

The original contributions presented in the study are included in the article/Supplementary Materials; further inquiries can be directed to the corresponding author.

## Footnotes

1 See https://spark.apache.org/docs/latest/streaming-programming-guide.html#upgrading-application-code

2 See https://github.com/rug-ds-lab/planning-dynamic-spark-supplemental
